# Sleep quality in cancer patients: a common metric for several instruments measuring sleep quality

**DOI:** 10.1007/s11136-024-03752-7

**Published:** 2024-08-05

**Authors:** Michael Friedrich, Thomas Schulte, Merle Malburg, Andreas Hinz

**Affiliations:** 1 Department of Medical Psychology and Medical Sociology, University of Leipzig, Leipzig, Germany; 2 Comprehensive Cancer Center Central Germany (CCCG), Leipzig, Germany; 3 Rehabilitation Clinic Bad Oexen, Bad Oeynhausen, Germany

**Keywords:** Sleep, Cancer, Common metric, Conversion, General population

## Abstract

**Purpose:**

Sleep problems are frequently observed in cancer patients. Multiple questionnaires for assessing sleep quality have been developed. The aim of this study was to present transfer rules that allow the conversion of the patients’ scores from one questionnaire to another. In addition, we anchored this common metric to the general population.

**Methods:**

A sample of 1,733 cancer patients completed the following questionnaires: Pittsburgh Sleep Quality Index, Insomnia Sleep Index, Jenkins Sleep Scale, EORTC QLQ-C30, and the sleep scale of the EORTC QLQ-SURV100. The methods for establishing a common metric were based on Item Response Theory.

**Results:**

The main result of the study is a figure that allows the conversion from one of the above-mentioned sleep scales into another. Furthermore, the scores of the questionnaires can be transferred to theta scores that indicate the position within the group of cancer patients and also to T scores that indicate the position in relation to the general population. The correlations between the sleep scales ranged between 0.70 and 0.85.

**Conclusions:**

The conversion rules presented in the study enable researchers and clinicians to directly compare single scores or mean scores across studies using different sleep scales, to assess the degree of sleep problems with regard to the general population, and to relate cutoff scores from one questionnaire to another.

**Supplementary Information:**

The online version contains supplementary material available at 10.1007/s11136-024-03752-7.

## Introduction

Cancer is a global public health hazard [1]. Worldwide, about 19.3 million new cancer cases were registered in 2020 [2]. Cancer patients experience a variety of limitations and symptoms. One of the most common and most distressing symptoms is sleep disturbance. Several reviews and meta analyses on cancer and sleep have been published in recent years [3–7], finding that up to 95% of cancer patients reported sleep problems.

Though sleep problems are also prevalent in the general population, the level of sleep problems is markedly higher among cancer patients than in the general population. Considering the 15 scales that are included in the quality of life questionnaire EORTC QLQ-C30, sleep problems ranked second after fatigue as aspects of quality of life that differed most between cancer patients and the general population [8]. However, oncologists often pay too little attention to sleep problems when treating cancer patients [9–11].

Multiple instruments have been developed for effectively and reliably measuring sleep quality. The most frequently used instrument appears to be the Pittsburgh Sleep Quality Index PSQI [12] that includes seven sub-domains. Other questionnaires, such as the Insomnia Severity Index ISI [13] or the Jenkins Sleep Scale JSS [14], comprise several items that have to be added up to a sum score without considering subscales. There are also instruments for measuring QoL or mental health that include one sleep item, e.g., the EORTC QLQ-C30 [15], the Patient Health Questionnaire PHQ-9 [16], and the GHQ [17].

Until now, it has nearly been impossible to transfer the results obtained with one of these sleep scales to another scale. This also implies that the cutoff scores for defining low or high degrees of sleep problems cannot be compared between the questionnaires.

A few studies used at least two sleep scales and reported their mean scores. Three studies with subjects suffering from sleep problems used the PSQI and the ISI and reported mean scores for both questionnaires [18–20]. Two recent examinations with cancer patients compared the results obtained using a multi-item questionnaire on sleep problems with a single-item assessment of sleep problems. The ISI was compared with the sleep item of the PHQ-9 [21], and the Jenkins Sleep Scale JSS was compared with the sleep item of the EORTC QLQ-C30 [22]. Such studies provide clues as to which values of one questionnaire correspond to particular values of another instrument. However, these studies were not designed to convert the results obtained with one instrument into scores of another one. Even if a certain degree of comparability can be derived from the mean scores of two instruments, it cannot be assumed that the relationship is linear when converting two measured values on different scales. This impedes the communication and interpretation of test results and consequently the implementation of research results in healthcare practice, e.g. in clinical monitoring or pooling datasets.

This problem of insufficient convertibility of instruments claiming to measure the same subject does not only exist in the field of sleep problems; it can be observed for nearly all constructs of physical and mental health. For some areas, attempts have been made to convert scores from one instrument to another. For example, a common metric for instruments measuring fatigue was presented [23] by establishing conversion rules for six different fatigue scales, based on a large sample of cancer patients. In addition to the conversion formulae, such analyses on a common metric also allow for a comparison of the reliability of the instruments [23]. Other areas for which such metrics have been calculated are depression [24–26], anxiety [27, 28], and pain [29]. A systematic comparison of several commonly used instruments for measuring sleep quality, including mutual conversions, has not yet been done.

The aims of this study were (a) to provide a table that allows conversion from one sleep assessment instrument to another, and (b) to compare these instruments concerning their reliability.

## Methods

### Sample of cancer patients

The study participants were recruited in an oncological rehabilitation clinic between July 2022 and June 2023. In Germany, most cancer patients are offered the opportunity to participate in rehabilitation programs to help restore their physical and psychosocial functioning. Inclusion criteria for this study were a confirmed cancer diagnosis, age of 18 years and above, sufficient command of the German language, and absence of severe cognitive impairment. Informed consent was obtained from the participants. A total of 2,250 consecutive patients were asked to participate, and 1,733 (77%) of them agreed to take part in the study. The Ethics Committee of the Medical Faculty of the University of Leipzig approved the study (approval number: 513/21-ek).

### Instruments

#### PSQI

The Pittsburgh Sleep Quality Index (PSQI) [12] is probably the most frequently used self-report instrument for assessing sleep quality. It consists of 19 items that are assigned to the following seven domains of sleep quality: subjective sleep quality, sleep latency, sleep duration, sleep efficiency, sleep disturbances, use of sleep medication, and daytime dysfunction. A global score of overall sleep quality can be calculated by adding up the single scores of these seven dimensions, resulting in a sum score ranging from 0 to 21. High scores indicate a high level of sleep problems. Global scores above 5 are generally used to indicate poor sleep. Using this criterion, 32% of the Austrian general population [30], 38% of the German general population [31], and 39% of the general population of Hong Kong [32] are poor sleepers. Normative scores of the PSQI are available [31].

#### ISI

The development of the Insomnia Severity Index (ISI) [13] was based on the diagnostic criteria for insomnia outlined in the Diagnostic and Statistical Manual of Mental Disorders (DSM-IV) and the International Classification of Sleep Disorders (ICSD). The instrument consists of seven items which cover sleep onset, sleep maintenance, early morning awakening, satisfaction level with current sleep pattern, interference with daily living, noticeability of impairment due to the sleep difficulty, and level of distress caused by the sleep problem. For each item, there are five response options (0–4), resulting in a sum score range from 0 to 28. The ISI sum scores can be divided into categories as follows: no significant insomnia (0–7), subthreshold insomnia (8–14), moderate insomnia (15–21), and severe insomnia (22–28) [13].

#### JSS

The Jenkins Sleep Scale (JSS) [14] is a four-item instrument for measuring common sleep problems: trouble falling asleep, waking up several times per night, trouble staying asleep, and waking up tired. For each item, there are six response options: not at all (0), up to 3 days a month (1), 4–7 days a month (2), 8–14 days a month (3), 15–21 days a month (4), and 22–31 days a month (5). This results in a sum score range from 0 to 20.

There is no generally accepted cutoff for poor sleep quality, though several proposals have been made, e.g., ≥ 12 for sleep problems [33], and 0–9 (none/ some), 10–14 (moderate), 15–20 (severe sleep problems) [34]. Normative values of the JSS are available [35].

#### EORTC QLQ-C30

The EORTC QLQ-C30 [15] was developed to measure quality of life in cancer patients. The instrument consists of 30 items and includes five functioning scales, a global health status/QoL scale, three symptom scales, and six single-item scales. One of these single items is the question “Have you had trouble sleeping?”. There are four response alternatives from 1 “not at all” to 4 “very much”. The scale is transformed to a range of 0 to 100, with higher scores representing more severe sleep problems. Here this single-item scale is referred to C30-SL. Normative values of the EORTC QLQ-C30, including the sleep scale, are available [36, 37].

#### EORTC QLQ-SURV100

The EORTC QLQ-SURV100 is a newly developed 100-item questionnaire for measuring QoL in cancer survivors [38]. One of its scales is the 4-item sleep quality scale, here called SURV-SL. The item which builds the C30-SL scale is included in this scale. As with the EORTC QLQ-C30, there are four response options per item, and the final sum score is converted into the range 0–100, with 100 indicating maximum sleep problems.

### Statistical analysis

The five sleep instruments were linked using a single group design. The common scale for item response theory (IRT) parameter estimates was obtained using concurrent calibration. That is, the parameters for all items are estimated simultaneously. We preferred this procedure over separate calibrations because it could not be assumed that each questionnaire would capture all commonality-constituting characteristics. Separate runs of parameter estimations for less comprehensive item sets (questionnaires with fewer items) are expected to produce biased estimates, resulting in a less accurate common metric. Concurrent calibration does not require separate runs [39] and it recovers the item parameters more accurately than separate calibration [40]. Estimation of the common latent trait was based on the graded response model. To reflect the structure of the data, we developed a bifactor model with one general factor and four specific factors to meet the two essential assumptions for calibrating items: Local independence and appropriate dimensionality [41].

To assess the degree of local (in-)dependence we examined the residual correlations matrix. Simulations showed that the residual correlations should be within a range of ± 0.3 around the average correlation [42]. The assumption of unidimensionality is supported, if two conditions are held. First, the degree of unidimensionality, as measured by explained common variance (ECV, percentage of common item variance that is due to the general factor) is greater than 0.8 [43], and second, if the dominant influence on item responses is a single latent factor, e.g., if coefficient omega hierarchical (omega_h, percentage of score variance due to the general factor) is greater than 0.8 [43]. Additionally, the percentage of uncontaminated correlations (PUC, one minus the number of correlations of items in specific factors per total number of correlations) is important to interpret ECV, since PUC moderates its interpretation: the greater the PUC, the less important is ECV in determining the degree of unidimensionality. Hence, if both ECV and PUC are greater than 0.7, the latent factor can be regarded as essentially unidimensional [43, 44].

Details on the evaluation of model fit are given in “Electronic supplementary material (Appendix)”, section “Model fit evaluation”.

To anchor the theta scores (mean = 0, standard deviation = 1) from the common metric of the patients to T scores (mean = 50, standard deviation = 10) of the general population (GP), we used data from a GP sample in which the PSQI was measured [31]. For this sample, we also used concurrent calibration and estimated an additional model that took into account the seven items (component scores) of the PSQI. In that way, we obtained two sets of theta scores (mean = 0, standard deviation = 1), one from the model for the patient sample and one from the model for the GP sample.

To link both groups (patients and general population), the theta scores from the GP sample were regressed on theta scores from the patients’ sample (separate calibration). This led to a regression formula, which then was transformed by multiplying with the standard deviation of 10 and adding the mean of 50 to allow a prediction of T scores of the GP based on theta values of the patients. More details on the method chosen to link both groups can be found in “Electronic supplementary material (Appendix)”, section “Separate calibration”.

Analyses concerning the common metric were carried out with R, version 4.4.0 [45] using the packages mirt, version 1.41 [46] for IRT analyses and ggplot2, version 3.5.1 to create graphics [47]. Further analyses were performed with SPSS, version 29 [48].

## Results

### Sample characteristics

A total of 1,733 cancer patients were included in the study. A proportion of 59.5% of the participants were women, and the mean age of the total sample was 56.0 years (SD = 14.5 years), see Table 1. The most frequent cancer type was breast cancer (32.3%).

**Table 1:** Sociodemographic and clinical characteristics of the sample (n = 1733)

	*n*	(%)
Sex		
Male	702	(40.5)
Female	1031	(59.5)
Age group		
18–39 years	254	(14.7)
40–49 years	276	(15.9)
50–59 years	417	(24.1)
60–69 years	464	(26.8)
≥ 70 years	322	(18.6)
Employment status ^a^		
Employed	996	(57.7)
Unemployed	63	(3.7)
Retired	589	(34.1)
Other	78	(4.5)
Education ^a^		
Elementary school (8–9 years)	356	(20.6)
Junior high school (10 years)	527	(30.5)
High school/university (≥ 11 years)	830	(48.1)
No formal qualification	13	(0.8)
Tumor localization		
Breast	560	(32.3)
Prostate	309	(17.8)
Gastrointestinal tract	290	(16.7)
Hematological	202	(11.7)
Female genital organs	108	(6.2)
Urinary tract	87	(5.0)
Melanoma	49	(2.8)
Thyroid / endocrine glands	38	(2.2)
Male genital organs	29	(1.7)
Others	61	(3.5)
Treatment		
Surgery ^a^		
No	177	(10.2)
Yes	1556	(89.8)
Chemotherapy ^a^		
No	882	(51.1)
Yes	843	(48.9)
Radio therapy ^a^		
No	952	(55.0)
Yes	779	(45.0)
Hormone therapy ^a^		
No	1247	(72.5)
Yes	473	(27.5)
Antibody therapy ^a^		
No	1452	(84.7)
Yes	262	(15.3)

^a^ Missing data not reported

### Mean scores and correlations among the sleep scales

The mean scores and the standard deviations of the scales are given in Table 2. The right part of Table 2 presents the correlations (Pearson’s r) between the scales. All correlations were 0.70 or above; the highest correlation was observed for the association between the ISI and the JSS (r = 0.85).

**Table 2:** Mean scores and correlations between the sleep scales

	M	(SD)	Correlations				
			ISI	SURV-SL	PSQI	JSS	C30-SL
ISI	12.6	(6.8)	-	0.77	0.81	0.85	0.78
SURV-SL	53.6	(28.1)		–	0.75	0.72	0.83
PSQI	8.3	(4.2)			–	0.72	0.74
JSS	11.3	(5.4)				–	0.70
C30-SL	54.8	(35.2)					–

*ISI*: Insomnia Severity Index; *SURV-SL*: symptom scale “sleep problems” of the EORTC QLQ-SURV100; *PSQI*: Pittsburgh Sleep Quality Index; *JSS*: Jenkins Sleep Scale; C30-SL: symptom scale “sleep problems” of the EORTC QLQ-C30; *M*: mean; *SD*: standard deviation

### Common metric of the sleep scales

The model on which the common metric was based included 19 items: seven items for the PSQI (one item for each dimension), seven items for the ISI, four items for the sleep scale of the EORTC QLQ-SURV100 including the item for the sleep scale of the EORTC QLQ-C30, and one item indicating the JSS. The four items of the Jenkins Sleep Scale (JSS) were parceled, that is, they were summed and included into the model as one item with values ranging from 0 to 20. This was necessary, because the instrument contains two items with nearly the same meaning: item 2 with “Wake up several times per night?” and item 3 with “Have trouble staying asleep […]” [14]. This resemblance represented a source of local dependence that could not be eliminated in any other way.

In the process of eliminating further sources of local dependence, we estimated a model for these 19 items with one general factor, marked residual correlations outside the interval of ± 0.3 around the average correlation, and hypothesized a specific factor that could explain the largest of the unusually high residual correlations independently from the general factor. Then we re-estimated the model as a bifactor model, marked the residual correlations again and hypothesized another factor, re-estimated the model and so on, until all correlations were within the interval. With this procedure, which is based on theoretical implications and statistical evidence, we identified 13 items as potentially problematic and hypothesized four specific factors as independent sources for an interference with the quality that the general factor measures. The following specific factors were modeled: S1 for external impairments to the restfulness of sleep (four items), S2 for external delays in falling asleep (three items), S3 for consequences of poor sleep quality (four items), and S4 for a scoring-induced dependence between two component scores of the PSQI (two items). These factors capture external influences which otherwise would have led to correlations between the respective items that cannot be explained by the common latent trait (general factor) of overall sleep quality. That is, establishing these four factors was necessary for establishing local independence. A diagram for the bifactor model as well as a substantive explanation for introducing the specific factors are presented in “Electronic supplementary material (Appendix)”, sections “Model diagrams” and “Rationale for specifying the specific factors”. The item parameters of the bifactor model are presented in “Electronic supplementary material (Appendix)”, section “Item parameters of the bifactor models”.

Regarding local independence, the mean residual correlation was 0.03. Applying the criterion of mean correlation ± 0.30 to justify the assumption of local independence, the residual correlations should lie in the interval from − 0.27 to 0.33. Since all residual correlations were in the range from − 0.27 to 0.27, local independence could be assumed. The bifactor model showed an ECV of 0.74 and a PUC of 0.91. Both values were above the cutoff of 0.7 and, therefore, indicate a sufficient degree of unidimensionality. The results on the evaluation of model fit are given in “Electronic supplementary material (Appendix)”, section “Model fit evaluation”.

**Fig.1:**
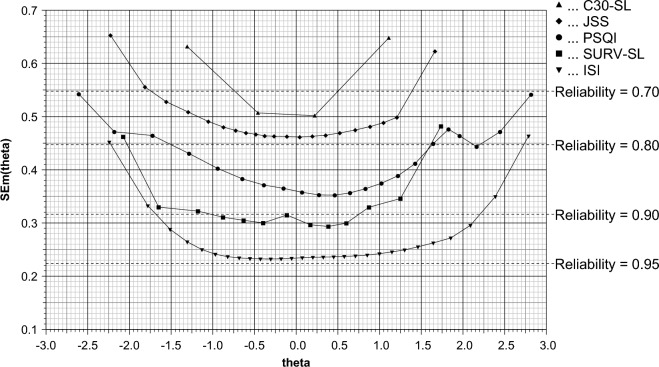
Measurement precision of the five instruments measuring sleep quality. *C30-SL*: symptom scale “sleep problems” of the EORTC QLQ-C30; *ISI*: Insomnia Severity Index; *SURV-SL*: symptom scale “sleep problems” of the EORTC QLQ-SURV100; *PSQI*: Pittsburgh Sleep Quality Index; *JSS*: Jenkins Sleep Scale; *SEm(theta)*: standard error of measurement, theta: estimated latent score for the patients’ population (mean = 0, standard deviation = 1)

Figure 1 illustrates the measurement precision in terms of standard error of measurement depending on the theta value (range: mean ± 3 standard deviations) for each of the instruments. The sleep scale of the EORTC QLQ-C30 consists of only one item with four response options. Therefore, this scale has only four data points in Fig. 1, while the JSS has 21 possible sum score outcomes (0–20) and, therefore, 21 data points.

The reliability is highest in the center of the figure, i.e., in the theta range from -1 to 1, while at the margins with very high or very low sleep problems the reliability is lower. Out of the five scales, the ISI was the most reliable instrument, with reliability coefficients above 0.90 in a wide range of theta values. The one-item sleep scale of the EORTC QLQ-C30 showed the lowest reliability scores, and the PSQI was in the middle range.

**Fig. 2:**
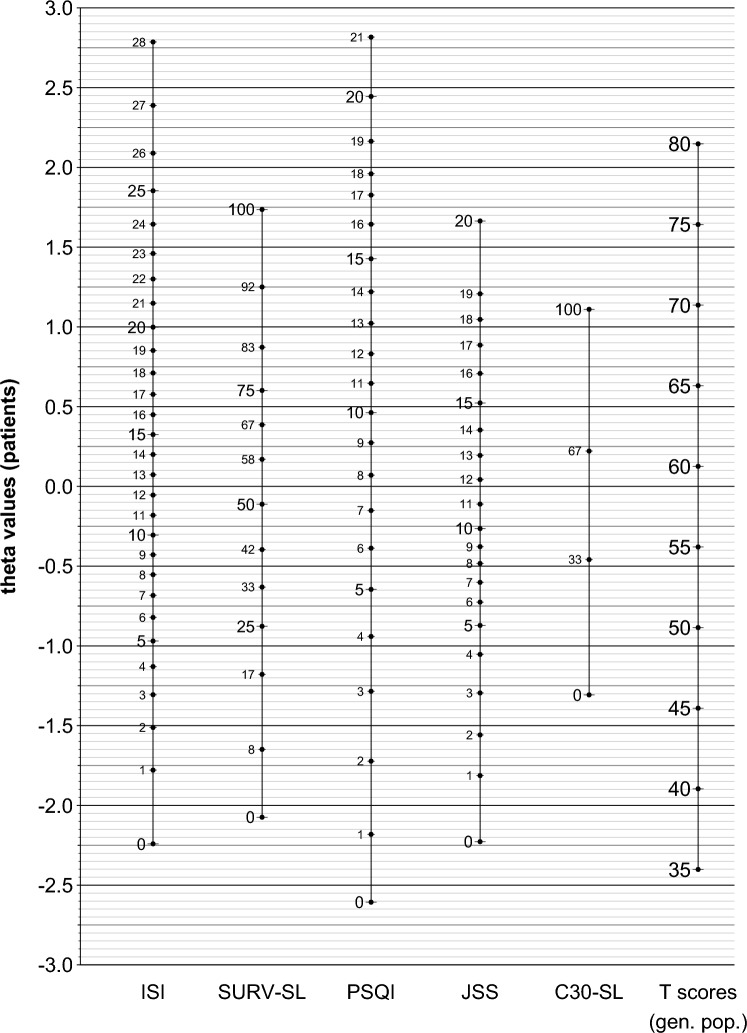
Common metric of sleep quality for five instruments. *ISI*: Insomnia Severity Index; *SURV-SL*: scale “sleep problems” of the EORTC QLQ-SURV100; *PSQI*: Pittsburgh Sleep Quality Index; *JSS*: Jenkins Sleep Scale; *C30-SL*: scale “sleep problems” of the EORTC QLQ-C30; T scores (gen. pop.): estimated T scores for the German general population (mean = 50, standard deviation = 10), theta values: estimated latent scores for the patients’ population (mean = 0, standard deviation = 1)

Figure 2 shows the common metric of the five instruments, anchored to the general population. The left axis indicates the theta values of the patients (mean = 0 and standard deviation = 1). Each data point in Table 3 shows the mean of theta values of the respective instrument with a specific score value. On all scales, higher scores indicate higher levels of sleep problems. For example, a score of 20 in the ISI represents a theta value of 1. In other words, patients with an ISI sum score of 20 report sleep problems that are one standard deviation above the mean of all patients. Scores of 12 and below in the ISI indicate sleep problems below the average of the patients. Moreover, an ISI score of 20 roughly corresponds to a PSQI score of about 13 and a JSS score of about 18.

**Table 3:** Theta scores of the sleep scales

Score	ISI	PSQI	JSS		Score	SURV-SL	C30-SL
0	− 2.24	− 2.61	− 2.23		0	− 2.07	− 1.31
1	− 1.78	− 2.18	− 1.81		8	− 1.65	
2	− 1.51	− 1.72	− 1.56		17	− 1.18	
3	− 1.31	− 1.28	− 1.30		25	− 0.88	
4	− 1.13	− 0.94	− 1.05		33	− 0.63	− 0.46
5	− 0.97	− 0.65	− 0.87		42	− 0.40	
6	− 0.82	− 0.39	− 0.73		50	− 0.11	
7	− 0.68	− 0.15	− 0.60		58	0.17	
8	− 0.55	0.07	− 0.48		67	0.39	0.22
9	− 0.43	0.27	− 0.38		75	0.60	
10	− 0.31	0.46	− 0.26		83	0.87	
11	− 0.18	0.65	− 0.11		92	1.25	
12	− 0.05	0.83	0.04		100	1.74	1.11
13	0.07	1.02	0.19				
14	0.20	1.22	0.35				
15	0.32	1.43	0.52				
16	0.45	1.64	0.71				
17	0.58	1.83	0.89				
18	0.71	1.96	1.05				
19	0.85	2.16	1.21				
20	1.00	2.44	1.66				
21	1.15	2.82					
22	1.30						
23	1.46						
24	1.64						
25	1.85						
26	2.09						
27	2.39						
28	2.79						

*ISI*: Insomnia Severity Index; *PSQI*: Pittsburgh Sleep Quality Index; *JSS*: Jenkins Sleep Scale; *SURV-SL*: symptom scale “sleep problems” of the EORTC QLQ-SURV100; *C30-SL*: symptom scale “sleep problems” of the EORTC QLQ-C30

### Anchoring the common metric to the general population

In addition to the theta scores calculated in relation to the sample of the cancer patients, the right axis of Fig. 2 indicates the standard scores with regard to the general population, expressed in terms of T scores (mean = 50, standard deviation = 10).

Anchoring of the common metric to the general population led to the following formula:

$${Tscore}_{GP}=9.89\times {theta}_{Patients}+58.76$$

According to this formula, patients with a theta score of about 0, i.e., patients with average sleep problems in the context of cancer patients, have a T score of about 59, i.e., about one standard deviation above the mean of the general population.

Table 3 presents the theta scores of the five scales that have already been illustrated in Fig. 2 in a numeric way. Regarding the EORTC QLQ-C30 sleep scale, there are only four possible scores: 0, 33, 67, and 100.

## Discussion

The central aim of this study was to calculate a common metric for five scales measuring sleep quality. Three of the five scales showed good reliability scores (above 0.80) in the middle range of the theta scores; about 68% of the participants fall in the range between -1 and 1. In addition to the usually reported reliability coefficients in terms of Cronbach’s alpha, Fig. 1 also illustrates how the reliability of the questionnaire depends on the range within the scale.

The one-item sleep scale of the EORTC QLQ-C30 showed the lowest reliability scores, which may be due to the low number of items. Despite the relatively large number of items and the frequent use of the PSQI, the reliability scores of this questionnaire were not optimal. This was also observed in other studies, with Cronbach’s alpha coefficients of 0.70 [49] and 0.77 [50]. One reason may be that the PSQI was designed to cover several distinct dimensions of sleep problems that are relevant in the context of sleep medicine, while other instruments such as the ISI or the JSS only include items that, more or less, ask for the subjective evaluation of sleep, which then results in higher correlations between the items, and, therefore, in higher alpha scores of these instruments.

Since the correlations between all scales were 0.70 and above, we believe that all scales are sufficiently suited to measure sleep problems in group comparisons.

Figure 2 illustrates the main result of the study, the common metric. This figure (or the corresponding scores given in Table 3) presents the opportunity to convert scale scores to theta scores and to convert the scores of one scale into scores of another scale.

The figure only presents integer scores that can be achieved by individuals. Mean scores obtained in samples of patients generally lie in between. In these cases, we recommend a linear interpolation. If, for example, the C30-SL mean score of a sample is 50, it lies between the scores of 33 and 67, which correspond to theta scores of -0.46 and 0.22. A linear interpolation can be performed in the following way:

$$theta\left(50\right)=-0.46+\frac{50-33}{67-33}\times \left(0.22-\left(-0.46\right)\right)=-0.46+ \frac{17}{34}\times 0.68= -0.46+0.34= -0.12$$

This score of -0.12 can also be roughly inferred from the position in Fig. 2. In addition to the conversion of single scores of mean scores, Fig. 2 also allows for the conversion of cutoff scores. The PSQI score of 5 is recommended as the threshold for high levels of sleep problems. According to Fig. 2 or Table 3, this would correspond to an ISI score of 7.3, which is at the threshold between the ISI ranges “no significant insomnia” and “subthreshold insomnia” [13].

A study with cancer patients used the ISI and the sleep item of the PHQ-9 [21] and reported mean scores of 12.4 ± 6.7 for the ISI and 1.65 ± 1.02 for the sleep item of the PHQ-9. According to Fig. 2, an ISI score of 12.4 is equivalent to a theta score of 0.0; therefore, the PHQ-9 sleep item mean score of 1.65 also corresponds to a theta of 0.0, and the standard deviation of 1.02 indicates that the theta range -1.0 to + 1.0 (one standard deviation below or above the mean) corresponds to mean scores of the PHQ-9 sleep item in the range from about 1.65 – 1.02 = 0.63 to 1.65 + 1.02 = 2.67. A further study used the ISI and the Athens Insomnia Scale (AIS) [50], and the authors reported mean scores of 10.54 ± 3.48 for the ISI and 9.83 ± 4.10 for the AIS, which also suggests including the AIS in the system of convertible sleep scales.

Figure 2 shows the assessment of sleep problems in the context of cancer patients (theta values on the left) and the general population (T scores on the right). The general population normative study of the JSS [35] reported a mean score of M = 3.83, which is slightly below the T score of 50 in Fig. 2. The general population mean score of the C30-SL (M = 15.7) [37] corresponds nearly exactly to the T score of 50. It can be concluded that the T-values of the general population at least do not systematically overestimate or underestimate the true mean values.

It is a matter of debate whether it is justified to use single sleep items from more comprehensive questionnaires such as the EORTC QLQ-C30 or the PHQ-9. Single items seem to be insufficient for individual diagnostics, but we think that the relatively strong correlations between the sleep items of the EORTC QLQ-C30 and the multi-item scales (r between 0.70 and 0.83) indicate a certain level of validity and justify the use at the group level. Another question is whether scales measuring daytime sleepiness such as the Epworth Sleepiness Scale ESS should also be included in such a common metric. In the Korean validation study of the ISI, this ESS was also used as a criterion of validity, and the PSQI also contains one scale (daytime dysfunction) that corresponds to daytime sleepiness. However, the correlation between the ESS and other sleep scales is low, e.g., r = 0.09 for the PSQI [51] and r = -0.28 for the ISI [18]. Therefore, we prefer not to include scales on daytime sleepiness in the common metric.

There are multiple research areas that are relevant in the clinical context and that could not be considered in this contribution. For example, we did not comment on sociodemographic and clinical factors that predict sleep quality [52], the relationship between sleep and quality of life [53], interventions to improve sleep quality [54] including the use of eHealth programs [55], and the relationship between subjectively assessed sleep quality and objectively measured parameters of sleep [56]. However, the conversion rules provided here also help qualifying the research on these topics by integrating the research results obtained with different instruments for measuring sleep quality.

## Limitations

Some limitations of the study should be mentioned. The sample of cancer patients might not be representative of all cancer patients. Patients with very severe health problems might feel unable to take part in the rehabilitation program, and, on the other hand, patients with few health problems may consider it unnecessary to regain fitness in the rehabilitation clinic. Therefore, the study might have focused more on patients with moderate health problems.

We used five sleep scales, although it would have been possible to select other scales, which could have consequences for the common metric. To match the results to the general population, we used a data set of only one questionnaire, the PSQI. Not only the sample of cancer patients, but also the sample of the general population may not be perfectly representative. People with a history of severe health problems were underrepresented in the general population sample [57], a problem that cannot be totally omitted in such general population examinations. The common metric was developed for cancer patients; it is a matter of future research to establish to what degree the conversion rules can also be applied to other patient groups or to the general population.

## Conclusions

Summing up, the results of this study provide a framework for integrating the research results obtained with different instruments in the context of measuring sleep quality.

## Supplementary Information

Below is the link to the electronic supplementary material.

Supplementary file1 (PDF 617 KB)

## Data Availability

The data underlying the common metric are available from the corresponding author upon reasonable request.
