# Supplementary material for: Sleep quality in cancer patients: a common metric for several instruments measuring sleep quality
Source: Qual Life Res. 2024 Aug 5;33(11):3081–91. doi: 10.1007/s11136-024-03752-7 (PMC11541315; doi:10.1007/s11136-024-03752-7)
Supplement: Supplementary file 1 — Supplementary file1 (PDF 617 KB) [file 11136_2024_3752_MOESM1_ESM.pdf]

Appendix: Details on model development

Sleep quality in cancer patients:
A common metric for several instruments measuring sleep quality

Michael Friedrich[1, 2*], Thomas Schulte[3], Merle Malburg[3], Andreas Hinz[1, 2]

Address:
[1] Department of Medical Psychology and Medical Sociology, University of Leipzig, Leipzig, Germany; [2] Comprehensive Cancer Center Central Germany (CCCG);[3] Rehabilitation Clinic Bad Oexen, Bad Oeynhausen, Germany; [*]Corresponding author

---

Contents

Model diagrams **2**
Figure A1 Diagram of the bifactor model for the patient population 2
Figure A2 Diagram of the bifactor model for the general population 2
Rationale for specifying the specific factors **3**
S1. External impairments to the restfulness of sleep (four items) 3
S2. External delays in falling asleep (three items) 3
S3. Consequences of poor sleep quality (four items) 4
S4. Scoring-induced dependence between two component scores of the PSQI 4
Model fit evaluation **4**
Table A1 Model fit 5
Separate calibration **6**
Table A2 Theta score estimations for the PSQI 6
Item parameters of the bifactor models **7**
Table A3 Item parameters of the bifactor model (patient sample), part 1 7
Table A4 Item parameters of the bifactor model (patient sample), part 2 7
Table A5 Item parameters of the bifactor model (general population sample) 8
References **8**

Model diagrams

To give a better idea of the models underlying this metric, we present here the model diagrams of the bifactor models for both samples (patients and general population).

Figure A1 Diagram of the bifactor model for the patient population

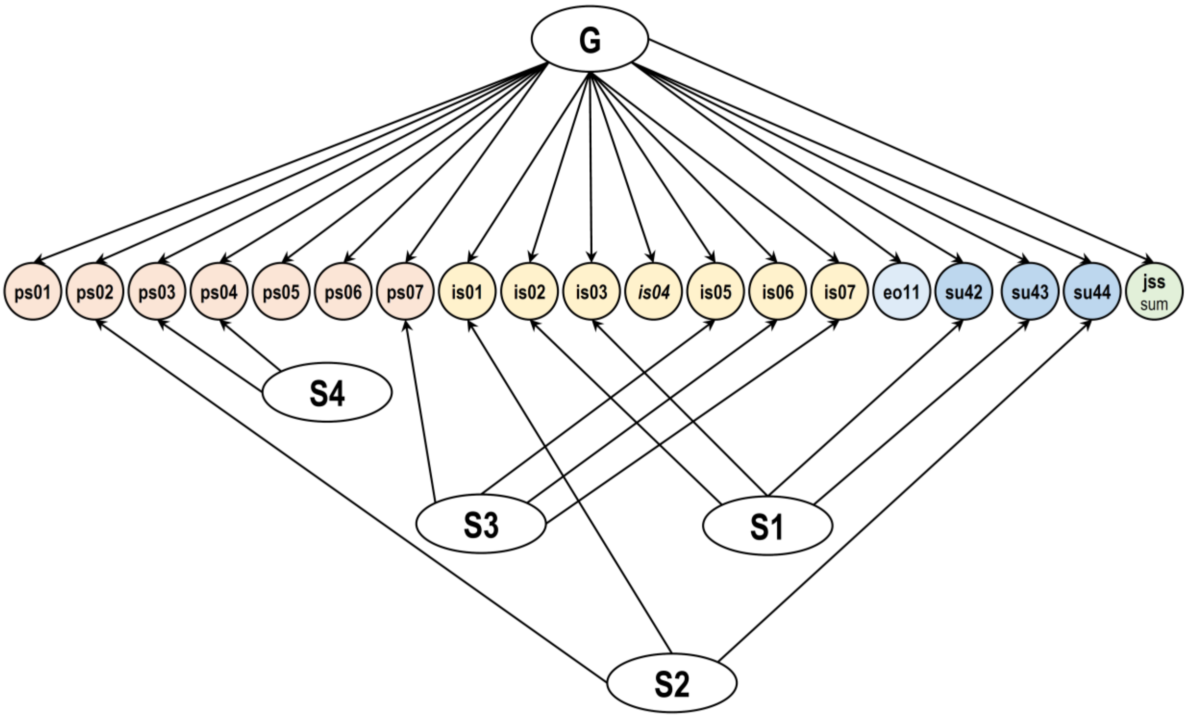

Abbreviations: **ps** denotes the items (component scores) from the Pittsburgh Sleep Quality Index, highlighted in red background color; **is** items from the Insomnia Severity Index, highlighted in yellow background color; **eo** items from the Questionnaires of the EORTC group, blue background color (**eo11** = symptom scale "sleep problems" of the EORTC QLQ-C30, **eo11 to su44** = symptom scale "sleep problems" of the EORTC QLQ-SURV100); **jss sum** denotes sum of the four items from the Jenkins Sleep Scale, green background color. **G** indicates the general factor. Specific factors: **S1** external impairments to the restfulness of sleep, **S2** external delays in falling asleep, **S3** consequences of poor sleep quality, **S4** scoring-induced dependence between two component scores of the Pittsburgh Sleep Quality Index.

Figure A2 Diagram of the bifactor model for the general population

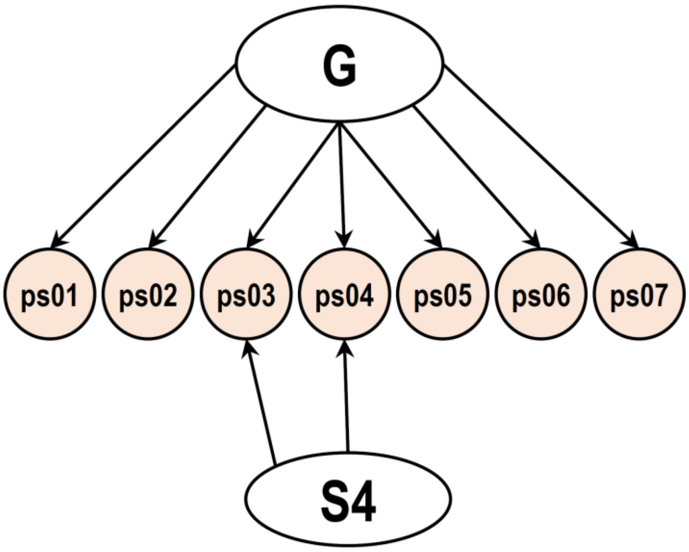

Abbreviations: **ps** denotes the items (component scores) from the Pittsburgh Sleep Quality Index, highlighted in red background color. **G** indicates the general factor. **S4** indicates the specific factor for a scoring-induced dependence between two component scores.

Rationale for specifying the specific factors

If the responses to the items from a common metric are correlated only through the general factor (gen), the item residuals should vary independently and their correlations should not exceed a specific threshold. Such a case indicates that the assumption of local independence is met. Residual correlations that exceed the threshold indicate local dependence, that is, they indicate another source of correlation between the items. Such sources can, for example, stem from similarities or ambiguity in the content or the response format of items, or different time frames etc. By modeling a specific factor (a factor that varies independently from the general factor and from other specific factors), it is possible to hypothesize an additional source of correlation between the respective items.
For the 19 items of the common metric of this study (see additional Figure A1), we identified four additional sources of correlation and modeled one specific factor for each of them. The descriptions of the items that are relevant for the respective specific factor are presented in the following four subsections.

S1. External impairments to the restfulness of sleep (four items)

- **is02**) Difficulties sleeping through the night.
- **is03**) The problem of waking up early in the morning.
- **su42**) Have you woken up during the night?
- **su43**) Have you woken up too early?

This factor comprises external impairments to the restfulness of sleep. They are no intrinsic part of sleep quality, because they indicate another relationship with everyday life. Like difficulties in falling asleep (specific factor S2), difficulties of sleeping through and of waking up indicate an attribute of sleep quality that depends on the respective everyday life environment. The specific factor controls for this dependence.

S2. External delays in falling asleep (three items)

- **ps02**) Component score for sleep onset latency in the Pittsburgh Sleep Quality Index. This item is a categorized index that is built from questions 2 and 5a:
  Q2) How long did it usually take you to fall asleep at night? (in minutes)
  Q5a) During the past month, how often have you had trouble sleeping, because you … Cannot get to sleep within 30 minutes? (response options: not during the past month, less than once a week, once or twice a week, three or more times a week)
- **is01**) Difficulties to fall asleep.
- **su44**) Have you had difficulty falling asleep?

This factor summarizes precursors of poor sleep, which prolong the phase of falling asleep. They are not an intrinsic part of sleep quality, because they rather indicate an attribute of sleep quality: the fit between everyday life environment and daily time of sleep. The everyday life environment confounds the measure of sleep quality, hence the factor controls for it.

S3. Consequences of poor sleep quality (four items)

- **ps07**) Component score for daytime sleepiness in the Pittsburgh Sleep Quality Index. This item is a categorized index which is built from questions 8 and 9:
  Q8) How often did you have difficulty staying awake, such as while driving, eating, or attending social events?
  Q9) Did you have trouble completing common daily tasks with enough drive?
- **is05**) How much do your sleep problems affect your daytime functioning (e.g., daytime fatigue, performance, concentration, memory, mood, etc.)?
- **is06**) How noticeable are your sleep problems and the deterioration in your quality of life to others?
- **is07**) How burdened are you by your current sleep problems?

This factor includes the consequences of poor sleep, such as difficulties staying awake, daytime sleepiness, or carrying out everyday tasks. These consequences are not an intrinsic part of sleep quality, because they rather reflect a relationship between sleep quality and everyday life of those affected. Because the same level of sleep quality can affect the daily functioning in different ways, depending on the respective everyday environment, these effects are biased indicators of sleep quality.

S4. Scoring-induced dependence between two component scores of the PSQI

- **ps03**) Component score for subjective sleep time in the Pittsburgh Sleep Quality Index. This item is a categorized index that is built from question 4:
  Q4) How many hours did you actually sleep per night? (This does not have to match the number of hours you spent in bed.)
- **ps04**) Component score for sleep efficiency in the Pittsburgh Sleep Quality Index. This item is a categorized index that is built from questions 1, 3, and 4:
  Q1) What time did you usually go to bed in the evening during the last four weeks? (hours, minutes)
  Q3) What time did it usually take you to get up in the morning? (hours, minutes)
  Q4) How many hours did you actually sleep per night? (This does not have to match the number of hours you spent in bed.)

This specific factor controls for a source of correlation that is due to a scoring-induced redundancy (similarity) of item content. Both component scores contain the number of hours of actual sleeping, hence they introduce a correlation, that cannot be explained by the latent factor for sleep quality (gen) alone.

Model fit evaluation

To indicate how the models reflect the structure of the data, we present the following fit coefficients for each estimated model: the limited information goodness-of fit test statistic M2 with df degrees of freedom, which is an equivalent to the Chi-squared test statistic in models of the classical test theory, the comparative fit index (CFI), the Tucker-Lewis-Index (TLI), the root mean square error of approximation (RMSEA), and the standardized root mean square residual (SRMSR).

Good model fit is indicated, if CFI or TLI are greater than 0.95, RMSEA < 0.06, or SRMR < 0.08 [1]. While RMSEA takes the complexity of the model into account, it depends on the number of items. Therefore, it is of interest when comparing different models. To assess if a model adequately fits the data, SRMSR is best used with SRMSR ≤ 0.05 [2].

Table A1 shows the model fit indices for both, the general population sample and the patient sample. For each sample, two models were estimated: One with the general factor only, which is denoted with the term “general”, and one containing the specific factor(s), denoted as “bifactor”. For both samples, the bifactor model shows adequate fit with $SRMSR_{patients} = 0.042$, $SRMSR_{general} = 0.036$. Taking the model complexity within both samples into account, the RMSEA of the bifactor model showed the better fit with a decrease of -0.06 in the general population and -0.08 in the patient population.

Table A1 Model fit

sample	model	M2 (df)	M2 / df	p-value	CFI	SRMSR	TLI	RMSEA (90%-CI)
**general population**	**general**	1875.5 (14)	134.0	<0.001	0.916	0.0716	0.874	0.119 (0.115 – 0.124)
	**bifactor**	412.4 (12)	34.4	<0.001	0.982	0.0359	0.968	0.060 (0.055 – 0.065)
**patients**	**general**	3969.5 (90)	44.1	<0.001	0.848	0.0777	0.816	0.158 (0.154 – 0.162)
	**bifactor**	819.8 (77)	10.6	<0.001	0.971	0.0421	0.959	0.075 (0.070 – 0.079)

Abbreviations: **general** indicates the model with only one general factor that assumes unidimensionality, **bifactor** indicates the model with (a) additional specific factor(s). **M2** limited information goodness-of fit test statistic, **df** degrees of freedom, **p-value** type-I-error probability of the M2 statistic, **CFI** comparative fit index, **SRMSR** standardized root mean square residual, **TLI** Tucker-Lewis-Index, **RMSEA** root mean square error of approximation, **90%-CI** 90% confidence interval of the RMSEA.

Separate calibration

For the linking between both groups, we used a separate calibration method. At first, we applied the Stocking-Lord (SL) method [3]. It uses the item parameter estimates from two separate calibrations to find a scale transformation formula based on the squared difference between both test characteristic curves [4]. These curves are a graphical representation of the sum scores of the test in dependence on the theta values (a.k.a. ability). Because of its robustness to effects of different item formats, this method is preferred over alternative methods [5]. But when we linked both groups, we obtained inconsistent results and we found that not every score of the Pittsburgh Sleep Quality Index (range 0 to 20) was observed in both samples. In the patient sample, the score 0 (best sleep quality) was missing and in the general population sample the upper scores from 19 to 20 (lowest sleep quality) were missing. Such a result is not unusual, even in large samples, as it is to be expected that patients report a poorer quality of sleep than respondents from the general population. Because this could be a reason for an inaccurate estimation of the theta values in these extreme sum score areas of the respective sample, we hypothesized that this might have led to the inconsistency. Hence, we excluded the theta scores of the unobserved sum scores from the calculations and decided to link the remaining theta scores (for the sum scores from 1 to 18) directly using linear regression. This method is similar to the SL method in that coefficients are determined that linearly link theta scores of each group. However, the link is no longer based on the item parameters from both groups directly, but on the theta estimates for which observations actually exist. The theta scores for each sum score are presented in table A2.

Table A2 Theta score estimations for the PSQI

PSQI sumscore	theta score PA	theta score GP	PSQI sumscore	theta score PA	theta score GP
0	-2.61	-1.76	11	0.65	1.52
1	-2.18	-1.17	12	0.83	1.72
2	-1.72	-0.74	13	1.02	1.91
3	-1.28	-0.41	14	1.22	2.11
4	-0.94	-0.12	15	1.43	2.30
5	-0.65	0.16	16	1.64	2.49
6	-0.39	0.42	17	1.83	2.69
7	-0.15	0.67	18	1.96	2.89
8	0.07	0.91	19	2.16	3.11
9	0.27	1.12	20	2.44	3.36
10	0.46	1.33	21	2.82	3.66

Abbreviations: **PSQI** Pittsburgh Sleep Quality Index; **PA** patient sample, **GP** general population sample.
Notes: Values in gray were excluded from the calculations for linking. The remaining values were used to determine the regression equation.

Item parameters of the bifactor models

Table A3 Item parameters of the bifactor model (patient sample), part 1

par	ps01	ps02	ps03	ps04	ps05	ps06	ps07	is01	is02	is03	is04	is05	is06	is07
**a1**	3.84	2.51	3.84	2.47	1.65	0.94	1.32	5.01	2.89	1.94	3.85	4.27	2.65	4.15
**a2**	0	0	0	0	0	0	1.38	0	0	0	0	3.02	2.44	1.86
**a3**	0	2.75	0	0	0	0	0	4.24	0	0	0	0	0	0
**a4**	0	0	0	0	0	0	0	0	0.09	0.92	0	0	0	0
**a5**	0	0	3.47	2.02	0	0	0	0	0	0	0	0	0	0
**d1**	7.18	3.61	0.62	1.66	5.63	-1.99	2.93	4.84	4.98	2.08	6.98	5.02	1.99	3.73
**d2**	-0.01	-0.22	-4.05	-0.77	-0.95	-2.44	-0.58	-0.37	2.09	0.20	2.62	0.83	-1.12	0.29
**d3**	-5.71	-2.85	-7.36	-2.59	-5.41	-3.11	-3.32	-4.87	-0.08	-1.58	0.21	-3.37	-4.45	-3.25
**d4**	.	.	.	.	.	.	.	-8.99	-2.24	-3.48	-3.72	-8.27	-8.10	-7.54

Abbreviations: **ps** denotes the items (component scores) from the Pittsburgh Sleep Quality Index, **is** items from the Insomnia Severity Index, **par** parameter, **a1 to a5** are slope parameters, **d1 to d4** are threshold parameters for response values.
Notes: Positive values are shown in green, negative values in blue.

Table A4 Item parameters of the bifactor model (patient sample), part 2

par	eo11	su42	su43	su44	jss sum
**a1**	3.33	2.69	18.50	2.68	3.30
**a2**	0	0	0	0	0
**a3**	0	0	0	2.07	0
**a4**	0	0.95	20.39	0	0
**d1**	3.59	5.11	25.36	2.41	8.63
**d2**	0.51	1.33	1.93	-1.01	7.30
**d3**	-2.48	-1.53	-18.93	-3.42	6.20
**d4**	.	.	.	.	4.94
**d5**	.	.	.	.	4.02
**d6**	.	.	.	.	3.35
**d7**	.	.	.	.	2.77
**d8**	.	.	.	.	2.30
**d9**	.	.	.	.	1.77
**d10**	.	.	.	.	1.41
**d11**	.	.	.	.	0.82
**d12**	.	.	.	.	0.11
**d13**	.	.	.	.	-0.47
**d14**	.	.	.	.	-1.16
**d15**	.	.	.	.	-1.82
**d16**	.	.	.	.	-2.60
**d17**	.	.	.	.	-3.39
**d18**	.	.	.	.	-4.11
**d19**	.	.	.	.	-4.78
**d20**	.	.	.	.	-5.52

Abbreviations: **eo11** one-item symptom scale "sleep problems" of the EORTC QLQ-C30, **eo11 to su44** four-item symptom scale "sleep problems" of the EORTC QLQ-SURV100, **jss sum** denotes sum of the four items from the Jenkins Sleep Scale. **par** parameter, **a1 to a4** are slope parameters, **d1 to d20** are threshold parameters for response values.
Notes: Positive values are shown in green, negative values in blue.

Table A5 Item parameters of the bifactor model (general population sample)

par	ps01	ps02	ps03	ps04	ps05	ps06	ps07
**a1**	3.78	1.70	1.41	3.35	1.66	1.30	0.88
**a2**	0	0	1.41	3.67	0	0	0
**d1**	4.79	1.20	-0.55	-0.69	0.28	-3.12	0.71
**d2**	-2.84	-1.27	-2.85	-4.44	-4.30	-3.80	-2.43
**d3**	-8.74	-3.34	-5.13	-7.30	-7.51	-4.60	-4.88

Abbreviations: **ps** denotes the items (component scores) from the Pittsburgh Sleep Quality Index, **par** parameter, **a1 to a2** are slope parameters, **d1 to d3** are threshold parameters for response values.
Notes: Positive values are shown in green, negative values in blue.

References

1. Revicki, D. A., Chen, W.-H., & Tucker, C. (2015). Developing item banks for patient-reported health outcomes. In S. P. Reise & D. A. Revicki (Eds.), Handbook of item response theory modeling: Applications to typical performance assessment (pp. 334–363, Multivariate applications series). New York, NY: Routledge Taylor & Francis Group.
2. Maydeu-Olivares, A. (2015). Evaluating the fit of IRT models. In S. P. Reise & D. A. Revicki (Eds.), Handbook of item response theory modeling: Applications to typical performance assessment (pp. 111–127, Multivariate applications series). New York, NY: Routledge Taylor & Francis Group.
3. Stocking, M. L., & Lord, F. M. (2016). Developing a common metric in item response theory. Applied Psychological Measurement, 7(2), 201–210. doi:10.1177/014662168300700208.
4. Lee, W.-C., & Ban, J.-C. (2009). A comparison of IRT linking procedures. Applied Measurement in Education, 23(1), 23–48. doi:10.1080/08957340903423537.
5. Kim, S., & Kolen, M. J. (2006). Robustness to format effects of IRT linking methods for mixed-format tests. Applied Measurement in Education, 19(4), 357–381. doi:10.1207/s15324818ame1904_7.
